# Omega-3 PUFAs as a Dietary Supplement in Senile Systemic Amyloidosis

**DOI:** 10.3390/nu15030749

**Published:** 2023-02-01

**Authors:** Lidia Ciccone, Susanna Nencetti, Armando Rossello, Lucia Barlettani, Nicolò Tonali, Paola Nieri, Elisabetta Orlandini

**Affiliations:** 1 Department of Pharmacy, University of Pisa, Via Bonanno 6, 56126 Pisa, Italy; 2 Interdepartmental Centre of Marine Pharmacology, University of Pisa, Via Bonanno 6, 56126 Pisa, Italy; 3 Research Centre E. Piaggio, University of Pisa, 56126 Pisa, Italy; 4 Centre National de la Recherche Scientifique (CNRS), Université Paris-Saclay, BioCIS, Bat. Henri Moissan, 17 Av. des Sciences, 91400 Orsay, France; 5 Department of Earth Science, University of Pisa, Via Santa Maria, 53, 56126 Pisa, Italy

**Keywords:** fatty acids, EPA, DHA, TTR, amyloidosis, diet, supplements, fish oil, omega 3, neuroprotection

## Abstract

Eicosapentaenoic acid (EPA; 20:5) and docosahexaenoic acid (DHA; 22:6), two omega-3 poly-unsaturated fatty acids (PUFAs), are the main components in oil derived from fish and other marine organisms. EPA and DHA are commercially available as dietary supplements and are considered to be very safe and contribute to guaranteeing human health. Studies report that PUFAs have a role in contrasting neurodegenerative processes related to amyloidogenic proteins, such as β-amyloid for AD, α-synuclein in PD, and transthyretin (TTR) in TTR amyloidosis. In this context, we investigated if EPA and DHA can interact directly with TTR, binding inside the thyroxin-binding pockets (T_4_BP) that contribute to the tetramer stabilization. The data obtained showed that EPA and DHA can contribute to stabilizing the TTR tetramer through interactions with T_4_BP.

## 1. Introduction

**Figure 1:**
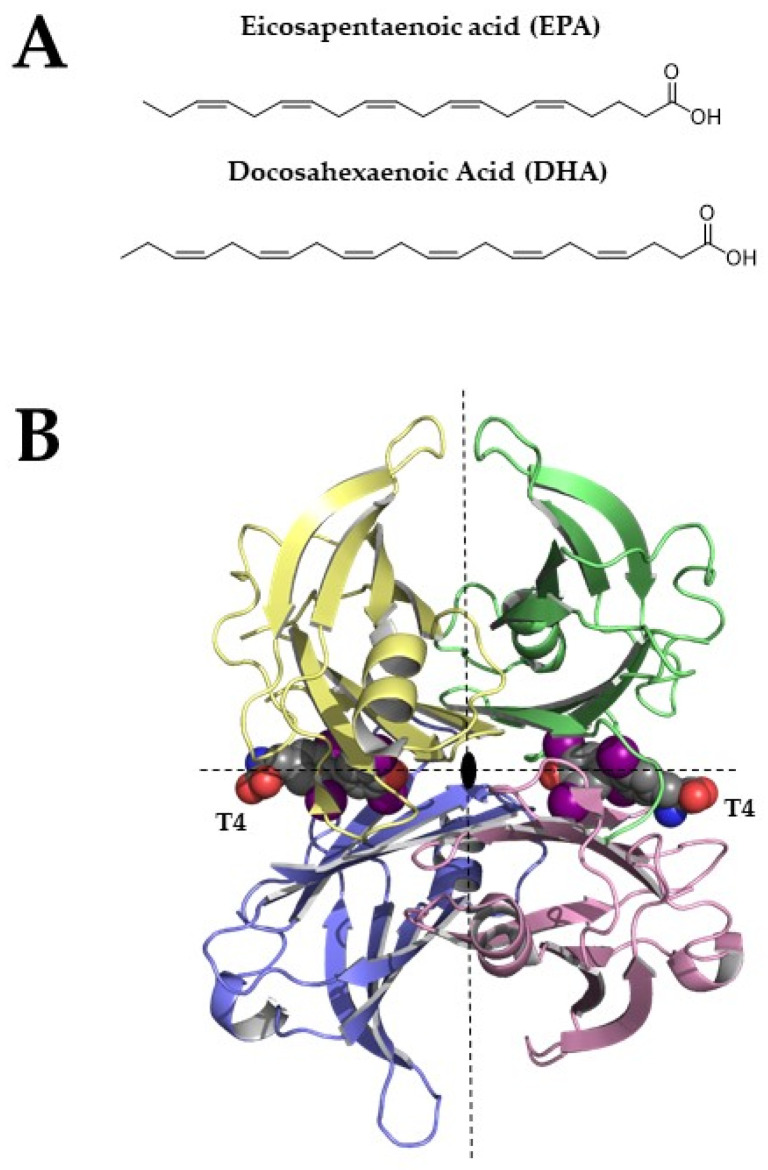
(**A**) Chemical structures of EPA and DHA (**B**) Tetrameric structure of TTR (pdb structure 1sn0), two thyroxine molecules bind the TTR-binding sites. Structural figures were made by PyMOL using our scripts [13,14,15].

Omega-3 poly-unsaturated fatty acids (PUFAs) including eicosapentaenoic acid (EPA; 20:5) and docosahexaenoic acid (DHA; 22:6) (Figure 1A), which are the main components in oil derived from fish and other marine organisms, when used as a dietary supplement, have several benefits for human health. Although the omega-3 PUFAs real role in major cardiovascular risks is currently controversial, they may help to reduce the development of coronary heart diseases by decreasing hypertriglyceridemia [1], blood pressure [2], and the chronic immune-inflammatory response that underlies atherosclerosis [3]. Other interesting effects of EPA and DHA may derive from their antioxidant activities [4,5]. Moreover, they have healthy effects on brain functions. Indeed, they showed to improve cognitive performances in terms of increasing learning, memory, and blood flow in the brain [6], and to ameliorate conditions for subjects with neurodegenerative disorders, such as Alzheimer’s and Parkinson’s diseases, (AD and PD, respectively) [7,8,9]. Some interesting evidence has also suggested a possible direct interaction between omega-3 fatty acids and proteins that are involved in neurodegenerative processes, i.e., β-amyloid for AD [10], α-synuclein in PD [11], and transthyretin (TTR) in TTR amyloidosis (ATTR) [12].

TTR, also known as human prealbumin, is a β-sheets-rich homotetrameric protein that is characterized by four equal monomers that are arranged together around a two-fold axis [16]. Each TTR monomer is characterized by two four-stranded antiparallel β-sheets and a short α-helix (Figure 1B). The two monomers assembled together form a dimer and the two dimers give life to the TTR tetramer. The tetramer is crossed by a channel that is divided into two different cavities, named thyroxine-binding sites (T_4_BP).

**Figure 2:**
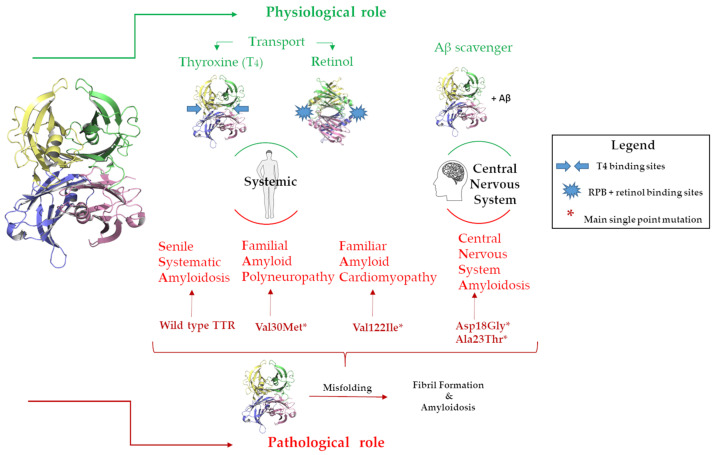
Graphic representation of the TTRs physiological and pathological roles in the body and in the CNS.

The circulating TTR is secreted by the liver, while the TTR in the cerebrospinal fluid is produced by the choroid plexus [17]. The acronym TTR holds the main protein’s physiological functions, namely transporter for thyroxine (T_4_) and retinol, the latter through binding with the retinol-binding protein (RBP) [18]. TTR is the second carrier of T_4_ in plasma and the first in cerebrospinal fluid (CSF) [19,20], Figure 2. Recently, TTR has been largely studied for its neuroprotective role in the central nervous system (CNS), where it has been demonstrated that TTR is able to bind Aβ favoring its scavenger from the brain to the liver [21,22,23,24,25]. Studies reported that TTR binds soluble Aβ peptides but also oligomers and fibrils contrasting the Aβ toxicity, decreasing the amyloid aggregates formation and preventing the fibril growth [26,27], Figure 2. Despite the several studies conducted, the precise binding mechanism of the interaction between TTR and Aβ is still unknown. However, experimental evidence supports the hypothesis that the tetrameric form of TTR is essential for binding with Aβ [26,28,29,30].

In contrast with this neuroprotective role, TTR possesses an intrinsic amyloidogenic potential related to its high level of the β strand [31,32,33]. Under pathological conditions, TTR undergoes a misfolding process that leads to the formation of protein aggregates and fibrils in the tissues leading to organ damage and dysfunction, inducing amyloidosis disease onset [34,35], Figure 2.

TTR can be responsible for several amyloidosis diseases, such as familial amyloid polyneuropathy (FAP), familiar amyloid cardiomyopathy (FAC), central nervous system amyloidosis (CNSA), and senile systematic amyloidosis (SSA) [36]. While SSA is related to wild-type TTRs (wt-TTRs), the others are associated with more than a hundred TTR point mutations [37]. One of the therapeutic approaches against TTR amyloidosis (ATTR) progression is the use of small molecules that are able to bind the T_4_BP, which contributes to the stability of the TTR tetramer [38]. Tafamidis was the first-in-class drug approved for the treatment of TTR amyloid cardiomyopathy, and it is still the only drug used in a clinical setting [39,40,41,42].

Several in vitro and in vivo experiments have shown that natural compounds are able to contrast the TTR tetramer desegregation [43,44,45,46]. A diet enriched with nutraceuticals, as supplements, is a potentially powerful tool to prevent or postpone the TTR misfolding process and amyloidosis [47,48]. In this context, we propose a preliminary investigation of EPA and DHA to evaluate if they can interact with the T_4_BP contributing to the wt-TTR tetramer stabilization.

## 2. Materials and Methods

### 2.1. In Vitro Studies

Reagents and solvents of analytical grade were purchased from Sigma-Aldrich (St. Louis, MO, USA). Diflunisal, tolcapone, and the binding of ANS (8-anilino-1-naphthalenesulfonic acid) were bought at Merck Life Science (Milano, Italy), while eicosapentaenoic acid (EPA) and docosahexaenoic acid (DHA) were purchased from Vinci-Biochem (Vinci, Italy).

#### 2.1.1. Turbidimetric Assay

Turbidimetric assay was carried out following the protocol previously described [49], in order to evaluate the TTR fibril formation. Lyophilized prealbumin and human plasma (wt-TTR) were purchased from Merck Millipore (Molsheim, France). Diflunisal, tolcapone, EPA, and DHA were dissolved in DMSO to reach a final concentration of 7.2 μM into the well. A solution of TTR was made using 10 mM phosphate buffer pH 7.6 (100 mM KCl, 1.7 mM EDTA) and was dispensed into wells of 96-well microplates (7.2 µM). The stock inhibitor solution or DMSO, for the negative control, was added to each well. The plate was incubated for 30 min at room temperature. Then, the acetate buffer (200 mM acetate at pH 4.4, 100 mM KCl, and 1.7 mM EDTA) was aliquoted in each well. The microplate was incubated at 37 °C for 72 h without stirring. After that time, the plate was vortexed, and the optical density was measured at 450 nm using a SPECTROstarNano (200–1000 nm) UV/Vis spectrophotometer [50]. All compounds were tested in triplicates and the percentage of fibril formation was calculated as previously described [51].

#### 2.1.2. Thioflavin T (ThT)

wt-TTR (7.2 µM), purchased from Calbiochem (EDM Millipore, cat.529577 lot: 2896620), was incubated overnight at 37 °C in 10 mM phosphate buffer (pH 7.0) in the presence of, or in the absence of 10 µM of diflunisal (positive control), EPA, and DHA. A total of 200 mM acetate buffer (pH 4.4) was successively added, and all the samples were incubated at 37 °C for 96 h (final concentration of TTR 3.6 µM). After incubation, amyloid fibril formation was assessed using a ThT-binding assay (10 µM ThT in 50 mM glycine buffer pH 9; TTR concentration at 0.045 µM). Bars are representative of the 7 dilution measurements from the same incubation vial. Values represent the mean ± the standard error (SEM). One-way ANOVA test has been performed for each condition in comparison to TTR alone.

#### 2.1.3. ANS Competitive Binding Assay

The competitive ANS-binding and its displacement by EPA and DHA were performed according to the procedure previously described [52]. TTR was incubated with ANS at room temperature for 15 min, in 96-well plates. Then, EPA and DHA at different concentrations (60 µM to 10 µM) were added. After 10 min, the plate was stirred, and the fluorescent emission spectra (400–540 nm) were recorded by exciting at 280 nm [44] using Molecular Devices SpectraMax Gemini XPS plate reader. Subsequently, the IC_50_ was determined, the ANS fluorescence was excited at 280 nm, and the emission was recorded at 470 ± 20 nm [53].

#### 2.1.4. Statistical Analysis

Data were presented as the mean ± standard error (SEM) of at least three independent experiments. All statistical analyses were performed using the GraphPad Prism software, version 7.0 (GraphPad Software Inc., San Diego, CA, USA). For the comparison of the experimental groups, a one-way ANOVA and a Turkey post-test were used. A *p*-value < 0.05 was set as statistically significant.

## 3. Results and Discussion

**Figure 3:**
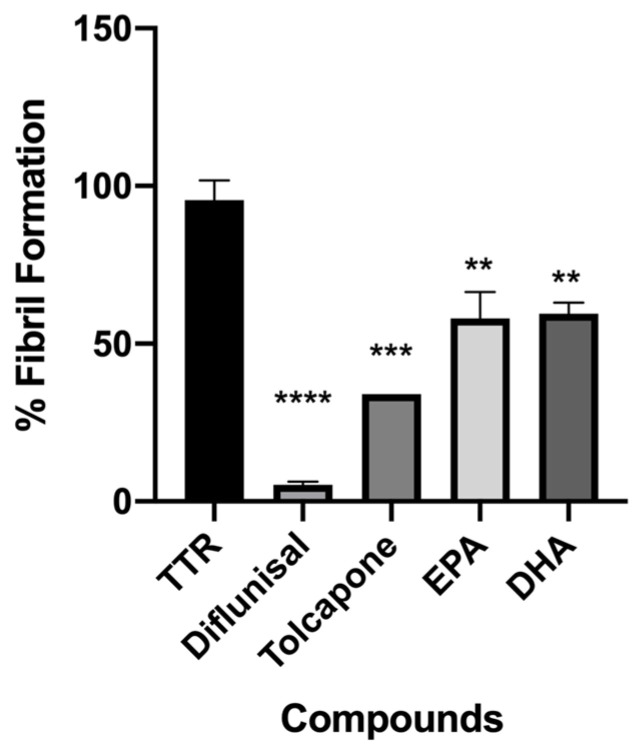
In vitro acid denaturation mediated of wt-TTR. The FF in absence of any inhibitor was assigned to be 100%. Results are shown as mean ± SEM. Statistical significance is calculated by one-way ANOVA test and Turkey post hoc test: * indicates significant difference vs. TTR (**** *p* < 0.0001, *** *p* < 0.001 and ** *p* < 0.01).

EPA and DHA were tested in vitro by the turbidimetric assay to evaluate their ability to inhibit the amyloid fibril formation (FF) of TTR. The results were reported as a percentage of the fibril formation, as shown in Figure 3. The ability of the two tested compounds to reduce the fibril formation was compared to diflunisal and tolcapone, which were used as a positive control [54,55]. TTR without an inhibitor was used as the negative control (100% of FF). The concentration used in the turbidimetric assay to screen the two compounds was 7.2 µM, which was twice the concentration of the TTR tetrameric form in plasma (3.6 µM) [56], and the data were recorded at 72 h.

Both fatty acids displayed a significant ability to contrast the fibril formation (36% EPA and 40% FF DHA), Figure 3.

**Figure 4:**
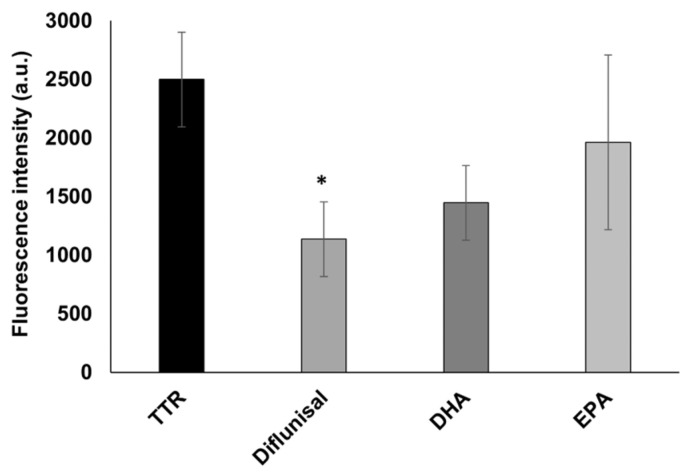
In vitro ThT fluorescence assay of wt-TTR in the absence or in the presence of diflunisal, DHA, and EPA. Values represent the fluorescence intensity mean ± SEM. The significance compared to TTR alone is assessed by one-way ANOVA test and Turkey post hoc test: * indicates significant difference vs. TTR (* *p* < 0.05).

The inhibition activity of EPA and DHA has also been assessed by the ThT fluorescence spectroscopy, to study the ability of both fatty acids to affect the TTR fibrillization by reducing the amyloid β-sheet content. Their activity has been compared to diflunisal as in the turbidimetric assay. As showed in Figure 4, diflunisal has been found to significantly (*p* < 0.05) reduce the β-sheet-rich fibrils, with a reduction of 54% of the ThT fluorescence intensity compared to TTR alone. A similar reduction with a value quite near significance (*p* = 0.06) has been found for DHA, which reduced the amount of β-sheet-rich structures by 42%, which is similar to the value found in the turbidimetric assay. However, no significant difference from TTR was observed for EPA, which induced a 22% fibril decrease. This could be explained by the fact that ThT might detect small protofibrils, which are not able to diffract well in the turbidimetric assay.

Looking at the chemical structure of the studied fatty acids that are characterized by a carboxylic group and an unsaturated aliphatic chain, we speculated that they are potentially able to interact with the internal cavity of the TTR-binding sites. In order to verify this hypothesis, the ANS displacement binding assay of EPA and DHA was performed on wt-TTR. ANS is a fluorophore that is capable of simultaneously binding both TTR-binding sites and inducing a high fluorescence signal [53]. When a studied compound binds to the TTR-binding sites and induces the quenching of the TTR-ANS fluorescence complex, it means that the molecule displaces ANS out of TTR cavities.

**Figure 5:**
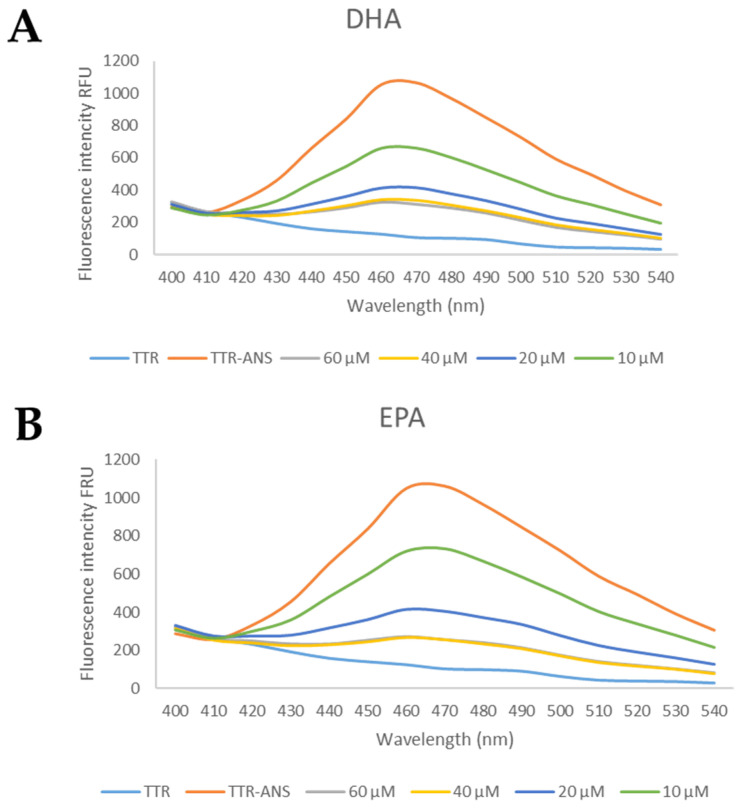
The general profile of ANS displacement binding assay for studied compounds. (**A**) The TTR-ANS complex was titrated with different concentrations of EPA and (**B**) of DHA.

Four concentrations of EPA and DHA were tested (60 μM, 40 μM, 20 μM, and 10 μM) and the preliminary results suggested that both fatty acids were able to displace ANS, Figure 5.

Starting from these data, several concentrations were investigated to calculate the IC_50_. Interestingly, EPA and DHA showed promising profiles as the TTR binder, with the IC_50_ of 13 μM and 12.1 μM, respectively, Table 1. These IC_50_ values are comparable with those of other natural compounds that are able to bind the TTR tetramer [48].

Comparing the results coming from the turbidimetric mediate test with respect to those of the ANS displacement binding test, we can assert that EPA and DHA interact with the T_4_BP and contribute to keeping TTR in its tetrameric form. These preliminary results suggest a possible beneficial use of these fatty acids as a dietary supplement in association with drugs for patients with TTR amyloidosis.

In AD patients, TTR levels are lower compared to healthy subjects [57,58], probably because TTR binds Aβ and favors its scavenger from the brain to the liver [23]. An in vivo study of old rats showed that the administration of EPA and DHA increased the expression of TTR gene in the brain by ten times [59]. Thus, they proposed that the regular consumption of fatty acids in the diet contributes to inducing TTR expression and that this can contribute to the clearance of Aβ from the brain. A few years later, EPA and DHA were used as supplements in AD patients, and also in this case, the TTR levels of expression increased significantly [60]. Therefore, for elderly people, the daily use of EPA and DHA can reflect an improvement in cognitive performance, as previously demonstrated [6], and also in positive effects mediated by TTR actions, inducing the TTR production and contributing to maintaining its tetrameric structure.

## 4. Conclusions

The data obtained from the present study, for the first time, show that the omega-3 PUFAs, EPA, and DHA, are able to contribute to contrasting in vitro TTR fibril formation through interacting with T_4_BP. This evidence is confirmed by the ANS competitive binding assay. Starting from these preliminary results, we suggest a possible beneficial use of PUFAs as a dietary supplement in association with drugs in elderly patients with senile systemic amyloidosis.

## Figures and Tables

**Table 1:** The IC_50_ values were calculated using ANS displacement binding assay.

Compound	IC_50_^a^ μM	R^2^
EPA	13.0 ± 0.08	0.9726
DHA	12.1 ± 0.07	0.9791

^a^ Mean ± SEM of three independent experiments.

## Data Availability

Not applicable.
